# Risk Factors Associated with Statin-Associated Muscle Symptoms in Patients Attending a Specialized Regional Lipid Clinic

**DOI:** 10.1155/2021/8882706

**Published:** 2021-03-19

**Authors:** M. K. Saeed, J. Shah, R. Damani, F. Rahman, P. Patel, P. Gupta

**Affiliations:** Department of Chemical Pathology & Metabolic Diseases, University Hospitals of Leicester, Leicester, UK

## Abstract

**Background:**

Statin-associated muscle symptoms (SAMS) are the major side effects reported for statins. Data from previous studies suggest that 7–29% of patients on statin had associated muscle symptoms. In the UK, there is a lack of corresponding data on SAMS and factors associated with the development of SAMS.

**Objective:**

This analysis is aimed at establishing the prevalence of SAMS and identifying major contributory risk factors in patients attending a lipid clinic.

**Methods:**

Clinical records of 535 consecutive patients, who visited the lipid clinic in the University Hospitals of Leicester, were studied retrospectively between 2009 and 2012. SAMS were defined by the presence of muscle symptoms with two or more different statins. Patients who reported muscle symptoms to statin with one or no rechallenge were excluded. The association of SAMS with clinical characteristics such as age and BMI, sex, smoking, excess alcohol, comorbidities, and medications was tested for statistical significance. A binomial logistic regression model was applied to adjust for risk factors significantly associated with SAMS.

**Results:**

The prevalence of SAMS was found to be 11%. On unadjusted analysis, the mean age of patients who had SAMS was significantly higher than those without SAMS (59.4 ± 10.5 years vs. 50.3 ± 13.4 years, respectively, *P* < 0.001). Nonsmokers were more likely to develop SAMS in comparison to active smokers (*P* = 0.037). Patients taking antihypertensive medications were more likely to develop SAMS (*P* = 0.010). In binomial logistic regression analysis, only age was positively and significantly associated with SAMS after adjusting for other risk factors (*β* = 0.054, *P* = 0.001).

**Conclusion:**

To the best of our knowledge, this study is the largest cohort of patients with SAMS in the United Kingdom. Our data suggest that the prevalence of SAMS is 11% and increased age is a risk factor associated with the development of SAMS in our cohort of patients.

## 1. Introduction

Hydroxy-methyl-glutaryl-coenzyme-A (HMGCoA) reductase inhibitors or statins play a significant role in the management of patients with increased cardiovascular disease (CVD) risk. They significantly reduce coronary events, strokes, and all-cause mortality [1]. However, evidence from nonadherence studies showed that almost half of patients starting statins discontinued their medication within a year [2].

Despite the relatively safe side effect profile of statins in the majority of patients, their use is limited in some patients due to a number of side effects. Statin-associated muscle symptoms (SAMS) are by far the major side effect reported. Data from previous studies suggest that 7–29% of patients on statin reported associated muscle symptoms [3], and patient registries reported up to 60% of statin users have experienced SAMS [4].

The mechanism by which SAMS are cause remains unknown.

SAMS is a spectrum of clinical entities that ranges from myalgia to rhabdomyolysis, and several clinical definitions were proposed for each clinical entity within the SAMS spectrum [5]. Numerous predictors were found to be associated with increased risk of SAMS such as increased age, female gender, Asian ethnicity, family history of myopathy, metabolic muscle disease, renal disease, hypothyroidism, diabetes mellitus, and a number of medications affecting statin pharmacokinetics [6]. In addition, genetic factors such as polymorphisms of the cytochrome P450 isoenzymes or drug transporters and certain inherited muscle diseases can increase susceptibility to SAMS ^6^. However, many of these aforementioned predictors were not replicable in subsequent studies with similar designs. Due to study design constraints, clinical trials were unsuccessful to replicate these predisposing factors [7].

Our analysis is aimed at identifying major contributory risk factors in our local patient population and at generally assessing determinants of population at risk of developing SAMS, using data from clinician-led services.

## 2. Methods

**Figure 1:**
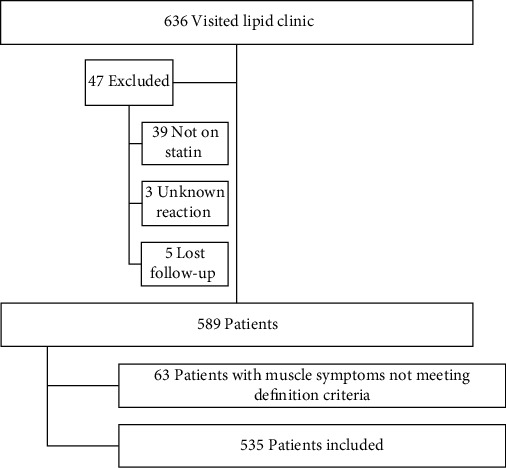
Flow chart of inclusion criteria.

Clinical records of 636 patients who visited the lipid clinic in the University Hospital of Leicester, Leicester, were studied retrospectively. Among the selected patients, 47 patients were excluded for different reasons (Figure 1). All participants were classified based on the presence or absence of SAMS.

All biochemistry tests were measured in accordance with standard laboratory techniques.

SAMS in general was defined by the presence of muscle symptoms after a total of two or more statin rechallenges. Patients who reported muscle symptoms to statin with one or no rechallenge were excluded (Figure 1).

An Independent two-sided *t*-test or chi-square test was used to determine the association of SAMS with clinical characteristics such as age, sex, BMI, smoking, excess alcohol, comorbidities, and medications. A binomial logistic regression model was applied to adjust for risk factors significantly associated with SAMS. *P* values less than 5% were considered significant in statistical tests used. The software package SPSS, version 25.0 (SPSS, Chicago, Illinois), was used for the statistical analysis.

Numerical codes were used to maintain the participants' privacy. The study was approved and registered as a clinical audit in accordance with the University Hospitals of Leicester local policies (audit number 10144) and in line with principles contained in the Declaration of Helsinki.

## 3. Results

The clinical and biochemical parameters of the first visit to the clinic were analysed in a total of 476 patients taking statin without muscle complications, and 59 patients were affected with statin-associated muscle symptoms as defined by inclusion criteria. The prevalence of SAMS, as defined above, was found to be 11%. The average age of patients who had SAMS was significantly higher than their non-SAMS counterparts (59.4 years vs. 50.3 years, respectively, *P* < 0.001).  Table 1 lists the main clinical characteristics of the subjects enrolled in the study.

To test the association of pharmacotherapy with a risk of SAMS, a qualitative comparison of both groups was performed. Patients who were taking concomitant antihypertensive medications were significantly more likely to have SAMS (Table 2).

Binomial logistic regression was performed to ascertain the effect of age, smoking status, and use of antihypertensive medications on the likelihood that participants have SAMS (Table 3). Of these three predictor variables, only increasing age was significantly associated with increased likelihood of developing SAMS.

## 4. Discussion

In this retrospective observation studies, we have shown that SAMS was diagnosed in 11% of our patients who were on statins. This is more or less similar to the 10.5% reported by the large observational PRedIction of Muscular Risk in Observational Conditions (PRIMO) study [8]. The percentage of statin user with SAMS ranged from 1.5 to 3% in most of the randomized controlled trials, possibly due to failure of selected population and/or study design to capture SAMS [7].

In this analysis, increased age was significantly associated with statin-associated muscle symptoms (Tables 1 and 3). In contrast to our findings, the PRIMO study showed no association of age on the likelihood of SAMS. However, the majority of observation studies, such as PRIMO, did not apply the clinical criteria of defining SMAS proposed by the EAS Consensus Panel [3], as well as the International Lipid Expert Panel [9], which include the inability to tolerate statin with one or more rechallenges. A systematic review and meta-analysis comprising of RCTs comparing the effect of statin monotherapy and placebo in adults older than 65 years showed little or no evidence in risks between treatment and placebo with myalgia, suggesting no evidence of increased risk of myopathy associated with increased age [8]. Leicester has a more ethnically diverse population compared to the locations of other studies; this might potentially account for the differences in outcome. Additionally, it was indicated in a metanalysis of a number of SAMS studies that the risk of myopathy and rhabdomyolysis was indeed increased in patients older than 65; however, the findings of the metanalysis suggested that it was not solely statin monotherapy, but the risk may generally increase in people over the age of 65. Furthermore, chronic conditions such as renal impairment, cardiovascular disease, and diabetes were associated with an increased overall risk of SAMS ^11^. In effect of STatins On Skeletal Muscle Performance (STOMP) trial, a randomized controlled trial which applied similar criteria for SAMS, the myalgia group had a significantly higher age than the entire study population [10]. Another study suggested that there is an association between age and the development of statin-associated rhabdomyolysis in particular, rather than SAMS as a spectrum [11]. We have also examined the relationship of polypharmacy in general and likelihood of developing SAMS, as a potential factor contributing to the association of increased age and SAMS. However, number of medications was not significantly associated with increased likelihood of developing SAMS. In regard to other demographic variables, we found no significant association of sex and BMI with the development of SAMS. This is consistent with results from previous studies [8, 10], although a few studies on statin-induced rhabdomyolysis and severe myositis showed association with sex, race, and small body frame and frailty [11].

The pharmacokinetics of statins plays an important role in potentiating the effect of statin on muscles and the pathophysiology of SAMS in general [12]. In our study, we examined the association of different medication classes and the likelihood of SAMS (Table 2). Only hypertensive medications and vitamin D supplements were significantly associated with higher chance of developing SAMS. However, this association (for antihypertensive drugs and SAMS) disappeared after adjusting it for age and smoking status in our regression model. Subsequently, we assessed the association of SAMS with an individual subclass of antihypertensive medications. There was no significant association between the individual antihypertensive subclasses and the risk of SAMS (Supplementary Table 1). Our analysis did not take into account the dose of the concomitant medications and therefore has limited inference in regard to cytochrome P450 inhibition and statin catabolism.

It is worth mentioning that in contrast to most SAMS-associated risk factor studies, this study used data generated by clinical assessment of specialized regional lipid clinic. The study design depends on a single time point when data was taken from the patient's first visit to the lipid clinic. This might not reflect the dynamics and change of these values when followed up in a cohort. Additionally, due to the scarcity of information, some variables (e.g., smoking) were taken as a binary variable. This may result in the limitation of data when adjusted in a binary logistic model of association.

In conclusion, we highlight age as an important parameter that influences the developing of SAMS in patients taking statin. Further studies need to be undertaken to investigate the aetiology of SAMS in the elderly. Analysing the combinatory effect of different risk factors can provide valuable information to stratify patients according to the predicted therapy responses. This will help prevent the development of SAMS and further optimize pharmacotherapy and cardiovascular risk management in these patients.

## Figures and Tables

**Table 1:** Clinical and biochemical characteristics of study subjects.

	All (*n* = 535)	No SAMS (*n* = 476)	SAMS (*n* = 59)	*P* ^∗^
Sex *n* (%)				
Female	214 (40)	190 (88.8)	24 (11.2)	0.910
Male	321 (60)	286 (89.1)	35 (10.9)	
Age (yrs)	51.3 ± 13.4	50.3 ± 13.4	59.4 ± 10.5	**<0.001**
BMI (kg/m^2^)	29.8 ± 5.9	29.7 ± 6.0	30.8 ± 4.9	0.177
Smoking status				
Active smoker (%)		36.8	19.4	**0.037**
Alcohol intake				
Excessive (%)		22.0	20.3	0.770
Hypertension				
Hypertensive (%)		38.1	50.8	0.059
Diabetes				
Diabetic (%)		23.5	22.4	0.798
Evidence of renal impairment				
Yes (%)		8.2	6.8	0.703
Evidence of liver impairment				
Yes (%)		8.4	3.4	0.176

Data are given as means ± SD. Abbreviations: BMI: body mass index. ^∗^*P* values represent the level of significance of the difference between patients who have developed SAMS and non-SAMS patients, calculated using an independent samples *t*-test. For the categorical variables, the chi-square test was used.

**Table 2:** Lists of concomitant medications of SAMS vs. non-SAMS patients.

	No SAMS (*n* = 476)	SAMS (*n* = 59)	*P* ^∗^
Number of medications	4.0	4.4	0.448
On antihypertensive medications			
Yes (%)	41.2	57.6	**0.016**
On antidiabetic medications			
Yes (%)	19.5	16.9	0.634
On pain killers			
Yes (%)	13.9	13.6	0.949
On aspirin/clopidogrel			
Yes (%)	21.6	25.4	0.508
On thyroid medications			
Yes (%)	11.8	10.2	0.718
On asthma medications			
Yes (%)	7.6	6.8	0.829
On antidepressant medication			
Yes (%)	22.1	15.3	0.229
On proton pump inhibitors			
Yes (%)	18.9	22.0	0.566
On other lipid-lowering agents			
Yes (%)	52.5	49.2	0.625

^∗^ *P* values represent the level of significance of the difference between patients who have developed SAMS and non-SAMS patients, calculated using the chi-square test. For the numerical, an independent samples *t*-test was used to test association.

**Table 3:** Model of adjusted significant parameters of SAMS.

	*B* (SE)	Odd ratio	95% confidence interval		*P*
			Lower	Upper	
Adjustment model^∗^					
Constant	-4.697 (0.886)				**<0.001**
Age	0.054 (0.015)	1.055	1.023	1.087	**0.001**
Antihypertensive medications	0.149 (0.378)	1.160	0.553	2.434	0.510
Smoking	-0.673 (0.450)	0.510	0.211	1.232	0.134

^∗^Binomial logistic regression model correcting for age, antihypertensive medications, and smoking status, the model was statistically significant, *P* value < 0.005. The model explained 11.5% of the variance in SAMS using Nagelkerke R2 methods.

## Data Availability

The anonymized patients' data used to support the findings of this study are available from the corresponding author upon request.

## Abbreviations

SAMS: Statin-associated muscle symptoms
HMGCoA: Hydroxy-methyl-glutaryl-coenzyme-A
UK: United Kingdom
BMI: Body mass index
CVD: Cardiovascular disease
CYP: Cytochrome P450
RCT: Randomized clinical trial
PRIMO trial: PRedIction of Muscular Risk in Observational Conditions trial
STOMP trial: Effect of STatins On skeletal Muscle Performance trial.

## Supplementary Materials

Supplementary Materials Supplementary Table 1: association of antihypertensive medications and likelihood of SAMS.
