# Supplementary material for: Risk Factors Associated with Statin-Associated Muscle Symptoms in Patients Attending a Specialized Regional Lipid Clinic
Source: J Lipids. 2021 Mar 19;2021:8882706. doi: 10.1155/2021/8882706 (PMC7997746; doi:10.1155/2021/8882706)
Supplement: Supplementary Materials — Supplementary Table 1: association of antihypertensive medications and likelihood of SAMS. [file 8882706.f1.docx]

Supplementary Table 1: association of antihypertensive medications and likelihood of SAMs

|  |  | No SAMS  (n=476) | SAMS  (n=59) | ***P**** |
| --- | --- | --- | --- | --- |
| **Calcium Channel Blockers** |  |  |  |  |
| Yes |  | 73 | 14 | **0.099** |
| No |  | 403 | 45 |  |
| **ACE inhibitors/receptor blockers** |  |  |  |  |
| Yes |  | 143 | 22 | 0.256 |
| No |  | 333 | 37 |  |
| **Beta blockers** |  |  |  |  |
| Yes |  | 91 | 16 | 0.147 |
| No |  | 385 | 43 |  |
| **Diuretics** |  |  |  |  |
| Yes |  | 64 | 8 | 0.981 |
| No |  | 412 | 51 |  |
| **Aldosterone antagonists** |  |  |  |  |
| Yes |  | 7 | 1 | 0.893 |
| No |  | 469 | 58 |  |
| **Alpha blockers** |  |  |  |  |
| Yes |  | 10 | 3 | 0.160 |
| No |  | 466 | 56 |  |
